# Glucose Driven Changes in Beta Cell Identity Are Important for Function and Possibly Autoimmune Vulnerability during the Progression of Type 1 Diabetes

**DOI:** 10.3389/fgene.2017.00002

**Published:** 2017-01-24

**Authors:** Gordon C. Weir, Susan Bonner-Weir

**Affiliations:** Joslin Diabetes Center, Harvard Medical SchoolBoston, MA, USA

**Keywords:** beta-cell biology, type 1 diabetes, autoimmunity, beta-cell identity, beta-cell function

## Abstract

This commentary explores the hypothesis that when autoimmunity leads to a fall of beta cell mass during the progression of type 1 diabetes (T1D), rising glucose levels cause major changes in beta cell identity. This then leads to profound changes in secretory function and less well-understood changes in beta cell susceptibility to autoimmune destruction, which may influence of rate of progression of beta cell killing.

## Commentary

There are important unanswered questions about what happens to pancreatic beta cells during the progression of type 1 diabetes (T1D). The process can evolve over many years from the appearance of beta cell autoantibodies, which is thought to result from injury by cytotoxic T cells, to the development of hyperglycemia, which is then typically followed by killing of the vast majority of beta cells. Some of the critical questions are: What happens to beta cell mass and function over time? What happens to beta cell vulnerability when its phenotype changes in response to rising glucose levels? What makes a beta cell a target? Why are only some cells being killed at any given time? How much regeneration occurs as the autoimmune process proceeds? These fundamental questions cannot be fully answered until we have a better understanding of the variations of beta cell identity that change over time.

## The beta cell as a moving target depending on cell identity

The importance of beta cell heterogeneity is becoming increasing obvious (Pipeleers, 1987; Bader et al., 2016; Dorrell et al., 2016). These variations in cell identity must be accompanied by changes in function and susceptibility to immune killing. Certainly there are differences depending on the age of a cell, such as new cells that come from self-duplication, cells that originate from neogenesis, or cells that might be considered middle-aged, senescent, or close to death. There must be a variety of other factors that contribute to heterogeneity. For example a beta cell that is adjacent to an alpha or delta cell may well differ because of paracrine influences unlike those in the center of the beta cell core. In spite of this well-documented beta cell heterogeneity, there is striking homogeneity of some aspects of function. For example, with even modest hyperglycemia the first phase insulin response to glucose is completely shut off in essentially every beta cell.

## Rate of decline of beta cell mass

Although, limited by our inability to measure beta cell mass as the disease progresses, we know that the rate of loss can differ greatly (Giannopoulou et al., 2015; Hao et al., 2016). A child developing T1D before 1 year of age clearly has a process of rapid beta cell destruction. In contrast another child may have autoantibodies at age two indicating that the process has begun; yet hyperglycemia may only appear 10 years later during adolescence. It is not uncommon for there to be a remission at that time with near normal insulin secretion usually measured as C-peptide. As a rough estimation, it may be that beta cell mass has fallen to 30–40% of normal with more insulin being secreted by whatever beta cell mass is remaining. We know that beta cell mass falls more rapidly once diabetes is diagnosed, most notably during childhood or adolescence. However, individuals diagnosed later, such as in their 20s or 30s will typically have a slower decline beta cell mass (Greenbaum et al., 2012). It is becoming more apparent that there is intensification of the autoimmune process as suggested by the increased appearance of both the types and increased titers of autoantibodies. The appearance of methylated insulin DNA in the circulation is a marker of beta cell death (Herold et al., 2015; Lehmann-Werman et al., 2016). During the progression of autoimmunity the levels of this marker fluctuate but are higher shortly before diagnosis, which suggests intensification of the attack. Another example of very rapid killing was shown when the pancreas of an identical twin without T1D was transplanted into the other twin with T1D; the beta cells were mostly destroyed in just weeks, while the rest of the pancreas presumably had no allograft rejection (Sutherland et al., 1989). We must be careful, however, about equating the titers of antibodies with intensity of beta cells killing because titers can fall while progression due to attack from CD8+ cells appears to continue (Martinuzzi et al., 2008).

## The distribution of islet inflammation

Examination of pancreases from people with T1D at various stages shows that few islets can be found with histological evidence of inflammation (Gepts, 1965; In't Veld, 2011, 2014; Campbell-Thompson et al., 2013). With some of these islets lymphocytes can sometimes be found just at the periphery, while in others the lymphocytes and other inflammatory cells can be found throughout the islet as beta cells are selectively killed. While the vast majority of islets have no evidence of insulitis, there are entire lobes completely free of visible inflammation (Gepts, 1965). Thus, depending on the stage of the process one finds normal looking islets and increasing numbers of pseudo atrophic islets containing mostly non-beta islet cells. This raises very interesting questions. Why do only a small number of islets show evidence of active autoimmune attack? Why should the islets of some lobes be completely spared at a point in time? How long can the surrounding lymphocytes maintain their position before entering the beta cell domains of the islet? And of course a very important question is: How long does it take from the appearance of lymphocytes around the islet until the beta cells are wiped out. In NOD mice, the killing takes place in a matter of weeks. Can the process have similar timing in human T1D?

There may be an explanation for the finding that some lobes have no evidence of islet inflammation. We know that pancreas weight increases along with the weight of other organs until growth stops shortly after adolescence (SBW, unpublished). Pancreatic growth occurs to a large extent by the formation of new lobes that include ducts, acini, and islets. Thus, it seems plausible that the older islets contained in older lobes are for some reason attacked first. Newly born cells as would be found in new lobes may go through a long period of maturation before they are recognized by the immune system.

Much is being learned about the cellular composition of inflammation in human islets. The cells surrounding the islets consist of CD8+ and CD4+ T cells as well as neutrophils and macrophages. As cytotoxic CD8+ T cells enter the islet, they are accompanied by B-cells (CD20+), which can be characterized as being either CD20Hi or CD20Lo. It is becoming apparent that a dominance of CD20Hi cells is associated with more aggressive and rapid killing (Morgan et al., 2014; Gomez-Tourino et al., 2016). A detailed summary of these cellular changes is beyond the scope of this chapter, but there is the concept of a “balanced autoreactive set-point” that refers to the balance between pro-inflammatory cells CD4+ cells secreting INF-gamma and/or IL-17 vs. CD4+ cells secreting IL-10 and or TGF-beta that have a regulatory suppressive effect (Gomez-Tourino et al., 2016). It seems entirely possible that rising glucose levels could change this balance.

## The relationship of hyperglycemia to beta cell function and susceptibility to autoimmunity

On one hand we know that beta cells exposed to even very slightly elevated glucose levels will undergo major changes in secretory function and phenotype (Jonas et al., 1999; Laybutt et al., 2003). Moreover, this change in phenotype, which becomes more marked as glucose levels rise, seems likely to be associated with changing vulnerability to autoimmunity.

With rising glucose levels, the changes in beta cell phenotype are probably very similar in all forms of diabetes whether T1D, T2D, or monogenic. The suggestion that insulin secretory function was altered by high glucose levels was first shown when rodent islets cultured at high glucose levels were found to have high basal insulin release and impaired insulin responses to acute increases in glucose levels (Andersson, 1974), a finding recently confirmed in cultured human islets (Henquin et al., 2015). This concept was best shown in the human *in vivo* studies of Brunzell et al. (1976), in which fasting glucoses only just above 100 mg/dl are associated with reduced first phase insulin responses to intravenous glucose; and when above only 114 mg/dl, the response is completely gone. These findings have now been replicated in many other studies. There are some studies, which conclude that β cell dysfunction can precede hyperglycemia (Weyer et al., 1999), but these studies did not rigorously exclude a contribution from mild dysglycemia. To conclude that glucose levels are truly “normal,” more is required than glucose tolerance tests and measuring fasting glucose levels. Similar results have been found in animal studies. A study by Laybutt et al. (2003) showed that after a 90% pancreatectomy in rats, many of the rats maintained what might look like “normoglycemia,” but dysglycemia was clearly present. The beta cells of these rats were found to have major changes in gene expression, which was almost certainly the result of the changes in glycemia, a process often called glucose toxicity (Weir and Bonner-Weir, 2013). Some argue that lipotoxicity and/or glucolipotoxicity contribute to these changes, but data showing adverse effects of free fatty acids or other lipid moieties on beta cells are largely based on *in vitro* studies, and the case for this occurring *in vivo* has only weak, if any, support.

Subjects with pre-T1D subjects have been found to have diminished first phase insulin responses in response to glucose shortly before obvious hyperglycemia develops. Originally studied by the Eisenbarth group, these subjects with reduced first phase insulin responses usually were found to have only modest dysglycemia (Ziegler et al., 1990) that did not even reach the definition of glucose intolerance This impairment of first phase release is very similar to what has been found as glucose levels rise during the progression to T2D. Thus, it seems highly likely that this beta cell abnormality in pre-T1D was caused by hyperglycemia. It is difficult to accept some alternative suggestions, such as this being due to either local inflammation or circulating cytokines because the insulitis in this stage of T1D is so patchy, and it is hard to imagine that circulating cytokine levels are high enough to produce such an effect. There are countless papers showing that inflammatory cytokines can kill beta cells *in vitro*, but we need to be careful about equating the doses used in these studies with what is actually occurring *in vivo*. It is logical to think that cytokines are responsible for β cell killing and dysfunction in islets that are full of lymphocytes, but these islets are few and far between.

## Beta cell changes driven by hyperglycemia could make them a more vulnerable target to autoimmunity

In 2008, Skowera et al. wrote, “It has long been speculated that β cells, stressed by the need to control blood glucose levels as diabetes develops, could become enhanced targets for the immune system” (Skowera et al., 2008). This group suggested that two naturally processed epitopes from the human preproinsulin signal peptide were increased by high glucose and served as targets for autoreactive cytotoxic lymphocytes. Thus, there are many ways in which rising glucose levels could influence the autoimmune process. The change in beta cell phenotype that occurs with mild dysglycemia could be associated with the appearance of a variety of new and important autoantigen derived epitopes (Delong et al., 2016; Gomez-Tourino et al., 2016). It is possible the glucose-driven endoplasmic reticulum stress or oxidative injury could be involved in the appearance of neoepitopes. Another change driven by glucose could be the release of chemokines such as CXCL10, MCP1, and MIP-1alpha by beta cells that might attract immune cells (Morgan et al., 2014). In addition there are a wide variety of other mechanisms involved in protection and vulnerabity that could be altered in this situation.

While it is tempting to think that phenotypic changes resulting from hyperglycemia should make the beta cell more vulnerable, some protective factors may be in play. When studying rat islets exposed to *in vivo* hyperglycemia following 90% partial pancreatectomy, we found that the beta cells were actually more resistant to streptozocin killing (Laybutt et al., 2002). Clearly this issue of beta cell vulnerability needs more study.

## Changes in beta cell vulnerability that are independent of hyperglycemia

When beta cells are attacked by autoimmunity, it makes sense that some would succumb more quickly than others. We know that β cells are heterogeneous even in normal pancreases, so this is yet another variable that can account for differential vulnerability. Simply put, there must be a normal ratio of vulnerable cells to resistant cells, and when autoimmunity progresses, the relative number of residual resistant cells should increase. However, the complexity and intensity of the assault appears to increase, so that even resistant cells are eventually killed. There has been great interest in the possibility that there are populations of beta cells that survive autoimmunity, this possibility being raised by finding that beta cells can virtually always be found in the pancreases of individual even decades after the onset of T1D (Keenan et al., 2010). These beta cells are very few in number and many of these individuals had no measurable circulating C-peptide. Evidence suggests that a low rate of neogenesis occurs throughout life (Bonner-Weir et al., 2010), so a more attractive explanation is that these residual beta cells are newly born and then killed once they develop a certain of level of maturity.

## How much beta cell regeneration might take place as autoimmunity progresses?

As was written by the pathologist Shields Warren in 1938: “The pancreas in diabetes is not simply the scarred field of an old battleground, but is the actual field of conflict. It does not submit without a struggle to injury, but endeavors to regenerate.” We know that human beta cells can replicate, particularly during childhood. We also know that adult human beta calls can replicate although we do not know to what extent. Ki67 marks cells that enter cell cycle and in pancreases obtained from adult subjects it is common to find that 0.05% of beta cells can be stained for Ki67. The big question is how many of these cells actually divide to produce two new cells? We also know that replication of human beta cells can be enhanced by hyperglycemia, so one would expect that as beta cell mass falls progressively due to autoimmunity and glucose levels start to rise, residual beta cells would be pushed to replicate, particularly in young subjects. A very instructive study published in 1907 reported the presence of islets with a large diameter in the pancreas of a 10-year-old boy who died of diabetes. The mean islet size was 240 × 188 μm whereas the mean diameter in five normal subjects was 157 × 146 μm. Because we know that increased demand from experimental insulin resistance leads to beta cell hyperplasia and increased islet diameter, it makes sense that the same process occurs as T1D develops.

The possibility that there is increased islet neogenesis is much less certain. With pancreas growth or regeneration, we think that all of the anatomical components of the pancreas increase, most obviously new ducts, acini, and islets. It is less apparent as to why islet neogenesis should be specifically stimulated in the absence of growth of these other pancreatic components. It has not yet been shown convincingly that increased glucose levels stimulate pancreatic duct cells to differentiate into new islet cells. Nonetheless, some provocative findings have been reported. In autopsy pancreases of subjects with insulin resistance, increased numbers of cells in ducts that are co-stained for insulin and cytokeratin have been described (Yoneda et al., 2013; Mezza et al., 2014). Moreover, in pregnancy, which is a state of insulin resistance, small collections beta cells have been found that could have originated from neogenesis (Butler et al., 2010). However, it is difficult to exclude the possibility that these result from hyperplasia of small extra-islet nests of beta cells.

## Hyperglycemia leads to reversible impairment of insulin secretion

The adverse effects of hyperglycemia on insulin secretion have been clearly shown in animal models and human T2D, and although less thoroughly studied in T1D, it is becoming clear that similar beta cell changes take place with all forms of diabetes (Weir and Bonner-Weir, 2013). The most striking defect is the loss of first phase insulin secretion to a glucose challenge. In spite of the presence of such a profound defect, glucose levels can remain in the state of glucose intolerance for many years. The glucose levels remain only slightly elevated because there is some second phase insulin secretion in response to glucose and the beta cells also receive parasympathetic stimulation from the cephalic activation, as well as receiving further stimulation from gut hormones such as gastrointestinal inulinotropic peptide (GIP) and glucagon-like peptide 1 (GLP-1). As beta cell mass falls and glucose levels rise further and insulin secretion becomes more impaired by glucose toxicity. It appears that secretion over 24 h can be reduced to only about 30% of normal, as deduced from the improvement of secretion that occurs in T2D when subjects are aggressively treated with insulin (Garvey et al., 1985). Thus, the glucose toxicity effect can be reversed when glucose levels are lowered, as is also very well shown when subjects with T2D normalize glucose levels by undergoing gastric bypass surgery (Polyzogopoulou et al., 2003).

The same kind of secretory changes can be seen in T1D, as strongly suggested by a study in Brazil that employed autologous bone marrow cells transplants with strong immunosuppression in an attempt to reset the immune system in subjects with new-onset T1D. An impressive number of remissions were obtained, which were associated with large increases in C-peptide secretion (Couri et al., 2009). It is highly doubtful that there was enough beta cell regeneration to account for this increase in secretion so relief of glucose toxicity appears to be the most logical explanation to account for a major part of the improved secretion. These findings have important practical ramifications, because as long as there are some significant number residual beta cells in recent onset T1D, or even with islet and pancreas transplantation, reduction of glucose levels with treatment will lead to improved endogenous insulin secretion.

## Summary

The main point of this commentary is to argue that when autoimmunity leads to a fall of beta cell mass in the progression of T1D, rising glucose levels cause major changes in beta cell identity. This then leads to profound changes in secretory function and less well understood changes in beta cell susceptibility to autoimmune destruction. This latter process should receive more attention.

## Author contributions

All authors listed, have made substantial, direct and intellectual contribution to the work, and approved it for publication.

### Conflict of interest statement

The authors declare that the research was conducted in the absence of any commercial or financial relationships that could be construed as a potential conflict of interest.
